# Body Composition and Cardiovascular Risk Factors in a Paediatric Population

**DOI:** 10.3390/children9050603

**Published:** 2022-04-24

**Authors:** Sonja Golob Jančič, Mirjam Močnik, Marjetka Švigelj, Nataša Marčun Varda

**Affiliations:** 1 Department of Paediatrics, University Medical Centre Maribor, Ljubljanska 5, 2000 Maribor, Slovenia; sonja.golob.jancic@gmail.com (S.G.J.); natasa.marcunvarda@siol.net (N.M.V.); 2 Medical Faculty, University of Maribor, Taborska 8, 2000 Maribor, Slovenia; svigelj.marjetka@gmail.com

**Keywords:** body composition, fat mass, clinical parameters, children

## Abstract

The aim of our study was to evaluate associations between body composition parameters and several clinical parameters. A total of 206 children and adolescents (120 male, 86 female) were prospectively included. Body impedance measurement was performed in all participants. During the hospital work-up, several clinical parameters such as anthropometric measurements and laboratory and ultrasound findings were obtained and correlated to body composition parameters. There was a significant association between body composition parameters and anthropometric measurements, systolic blood pressure, insulin levels, serum creatinine, urate, liver function tests, triglycerides, cholesterols and apolipoproteins, homocysteine, vitamin D and proteins in 24-h urine. Body composition differed by gender, between participants with and without hepatic steatosis and between patients with and without left ventricular hypertrophy. Interestingly, body composition did not correlate with diastolic blood pressure, pulse wave velocity and intima media thickness. This study showed that several clinical parameters are associated with body composition in children. Obesity and body composition play an important role in the development of other cardiovascular risk factors and are not dependent on fat mass alone, and the latter might be used for cardiovascular risk determination.

## 1. Introduction

Body composition measurement using bioelectrical impedance is being increasingly implemented in clinical work. It is a non-invasive, painless procedure that permits the estimation of the following body compartments: fat mass, fat-free mass and total body water [1].

A portable device generates a painless low-amplitude electrical current through cables connected to electrodes or to conducting surfaces placed in contact with the skin, permitting the measurement of resistance (R) and reactance (Xc) [1]. These measurements applied to mathematical equations can predict body compartment volumes. Phase angle (PA) is an indicator based on R and Xc and has an important prognostic role [2]. R comes from body fluids (extra and intracellular fluids behave as resistive components, and R is inversely proportional to fluid volume), and Xc arises from cell membranes. In many studies, Pa has been used as an indicator of nutritional status, disease prognosis, mortality and cellular vitality [3]. It has also been recommended as a prognostic tool in clinical settings. Reference data have already been published for both children and adults [4].

In children, body composition measurement is an important predictor of cardiometabolic diseases and can be used in hypertension risk stratification [5]. Childhood obesity increases the risk of cardiovascular morbidity, and bioimpedance analysis (BIA) is a useful tool in fat mass determination. It can be more precise than triceps skinfold thickness and body mass index (BMI) in epidemiological studies [6]; however, some studies indicate that in severely obese children, the percentage of fat mass can be underestimated [7]. Reassuringly, some studies have shown that BIA alone can be used as a substitute (or alternative) measure of fat-free mass in children [8].

The aim of our study was to evaluate the association between body composition parameters, especially fat mass, and other possible clinical predictors of cardiovascular risk to identify the role of BIA in children with hypertension. A wide spectrum of clinical data was collected prospectively, all associated with children’s cardiovascular risk and nutritional status. Therefore, we aimed to determine the correlation between body composition measurement and several clinical parameters in children and adolescents such as anthropometric measurements, laboratory or ultrasound findings in order to provide additional insight into the effect of body composition, particularly fat mass, on cardiovascular risk factors.

Several laboratory markers associated with obesity and nutritional status were included. In recent years, obesity has been increasingly recognised as a chronic pro-inflammatory state with increased inflammatory markers, such as interleukin 6 and can lead to an increment of C-reactive protein [9], which is the reason for the wide spectrum of laboratory data gathered, including leukocyte count. Inflammation is related to atherosclerosis, metabolic syndrome, insulin resistance and diabetes mellitus, which are all contributors to cardiovascular diseases.

Pulse wave velocity (PWV) and intima media thickness (IMT) are known cardiovascular risk markers. They were additionally determined in our cohort of children to evaluate their association with body composition. PWV is a marker of arterial stiffness that is associated with increased mortality in the elderly [10]. In children with obesity or hypertension, it is independently correlated to age and gender [11]. IMT is associated with obesity in children and is considered to be a noninvasive marker for early atherosclerotic changes [12].

## 2. Materials and Methods

A total of 206 children and adolescents (120 male, 86 female) were prospectively enrolled in this study. The study was approved by the institutional ethics committee (UKC-MB-KME-15/18) on 28 March 2018. All participants or their parents (legal guardians) consented to participation, and this was recorded on a written consent form. Children with hypertension, regardless of their obesity status, were included. They were enrolled during their hospital diagnostic work-up for hypertension management. Measurements of body composition using BIA were performed from October 2019 to October 2020 with the same protocol using a Nutrilab Bioimpedance device Akern 2016 with Biatrodes, Akern electrodes. Subjects were fasted with an empty bladder and in a supine position. BIA is a simple, non-invasive, bedside method of body composition assessment. The measurement environment was kept consistent: electrode position, no jewelry between electrodes, no exercise within 12 h and no caffeine or alcohol consumption within 24 h [13]. The parameters that were calculated using the manufacturer’s software (Bodygram Plus 1.2.2.8, Akern) included: PA, body cell mass (BCM, in kg/m), fat-free mass (FFM, in kg/m), fat mass (FM, in kg/m), total body water (TBW, in L/m) and extracellular water (ECW, in L/m). PA represents a mathematical relationship between R and Xc. BCM is the living, protein-based, metabolically active tissue in the body. FFM is defined as the difference between body weight and fat mass. FM is a storage compartment for adipose tissue filled with glycerol and fatty acids. TBW includes water that is inside and outside the cells and varies with age, gender and muscle mass. ECW presents the fluid outside of cells (vessels, lymphatics) [14].

Additionally, other cardiovascular risk factors were checked, and additional data were collected. Participants’ height and weight were measured by trained personnel with calibrated gauges following the same procedure. BMI was calculated as a person’s weight in kilograms divided by a person’s square height in m^2^. Subsequently, children and adolescents below the 90th percentile for their age and sex, according to the World Health Organization percentile chart, were marked as normal weight, and those above the 90th percentile were marked as overweight. Waist circumference (WC) was measured at the slimmest part of the waist below the lower ribs and above the navel. Hip circumference (HC) was measured around the fullest part of the childrens’ hips. Average daily blood pressure was determined with 24-h blood pressure measurement (SpaceLabs device) that measured blood pressure every 20 min during the day and every 30 min during the night. PWV was determined with the applanation tonometry technique. IMT and the presence of hepatic steatosis and left ventricular hypertrophy were determined using ultrasound techniques. Furthermore, laboratory work-up included an oral glucose tolerance test, the levels of insulin during the oral glucose tolerance test taken at fasting level and after 2 h of the beginning of the test, thyroid hormone levels, blood count, C reactive protein, urea with creatinine, urate, liver enzymes, lipidogram, cystatin C, lipoprotein (a), apolipoprotein A1 and B, urine albumin and creatinine ratio, homocysteine, serum vitamin D level, vitamin B12, folate, zinc and selenium.

SPSS Statistics (IBM, Armonk, NY, USA, version 22) was used for statistical analysis with basic descriptive statistics used for cohort presentation with all variables included. The Mann–Whitney test was used for the comparison between gender and weight categories for all variables included in the study, because not all variables were normally distributed. Pearson correlation test was used for correlations between body composition parameters and numerical variables. However, some variables were not numerical, such as the presence of hepatic steatosis. For these, an independent samples *t*-test for the grouping variable and body composition parameters was used. In the latter case, variables were normally distributed, and therefore, a parametric test was used.

A Pearson correlation test was used with an r factor of less than 0.3 indicating mild correlation, 0.3–0.7 indicating moderate correlation and more than 0.7 indicating strong correlation. *p* < 0.05 has been considered statistically significant. For multiple correlations, a Bonferroni correction has been calculated as the number of correlations divided by 0.05, e.g., the significance.

## 3. Results

Our population and obtained results are descriptively presented in Table 1 with subdivision by gender and obesity level, e.g., BMI over 90th percentile for age. Results are presented as median ± interquartile range, as some of the variables were not normally distributed according to the Kolmogorov–Smirnov and Shapiro–Wilks tests; however, the skewness was between +1 and −1. Statistically significant differences between groups are highlighted.

The main results of the correlations between body composition and numerical clinical parameters are presented in Table 2. Due to the numerous correlations, a Bonferroni correction has been calculated, setting the significance at *p* < 0.0002. The correlations reaching the corrected significance are marked.

The full blood count and biochemistry results were also compared, but no significant findings were noted; therefore, the data are not shown.

Next, we compared body composition data according to gender, the presence of hepatic steatosis, left ventricular hypertrophy and pathological oral glucose tolerance test with independent samples *t*-tests. The results are presented in Table 3. Body composition parameters were higher in males, though the average age between both groups did not differ significantly (13.4 years in males and 12.3 years in females with *p* = 0.062). Additionally, the body composition parameters were higher in patients with both hepatic steatosis and left ventricular hypertrophy.

Multivariate regression analysis was performed for all BIA parameters showing the importance of several factors. The model for FM included clinical parameters that were found significant in Pearson correlation tests after Bonferroni correction. The best model for FM is presented in Table 4. The adjusted R^2^ was 0.780 with *p* < 0.001.

## 4. Discussion

The prevalence of obesity and other cardiometabolic risk factors has increased in recent years [15]. BIA is a valid and helpful technique for monitoring childhood overweight and obesity [16]. The aim of our study was to evaluate the association between body composition parameters, especially FM, and other possible clinical predictors of cardiovascular risk.

Anthropometric parameters are usually used while assessing overweight status and obesity. Commonly, BMI has been used as a definition of obesity; however, several anthropometric parameters should also be included. Abdominal fat mass has been associated with multiple cardiovascular risk factors in children [17]. In previous studies, WC measured at the midpoint between the last rib and the iliac crest has the best correlation with the percentage of body fat measured with BIA [18]. In our study, body composition parameters correlated with all anthropometric parameters, namely height, weight, waist and hip circumference. BMI correlated the most with FM. Weight itself correlated significantly with BCM and FFM to a similar degree as FM, thus indicating the promotion of non-fat tissue growth in obesity as well. Assuming that children with obesity develop a certain grade of insulin resistance, which is proven with elevated insulin levels in their blood [19], we can conclude that increased BCM and FFM along with FM could be the consequence of insulin’s anabolic function [20]. WC showed more links with FM than HC, thus confirming previous studies [18].

Obesity is a well-known risk factor for arterial hypertension [21]. In our study, FM and other body composition parameters showed a significant connection only with systolic blood pressure but not with diastolic. This is in contrast to some previous studies, where correlations were found for both systolic and diastolic blood pressure [21]. Many children in our study had normal weight; therefore, we additionally separated the group into obese and non-obese children (BMI > 25 kg/m^2^ at the age of 18 years) according to Burniat et al. [22] and performed an analysis between FM and diastolic pressure separately in the group with obesity (data not shown), but we could not prove a significant correlation. In a large study by Chiolero et al., hypertension in schoolchildren was mainly isolated to systolic pressure and associated with excess body weight [23], which supports our findings.

PWV and IMT did not show a significant connection with FM and PA, but they did with other body composition parameters. Again, we performed a separate analysis for children with obesity (data not shown), but no findings were significant.

The level of insulin in the blood correlated significantly with FM, but not with other parameters. Obesity has already been shown to be associated with hyperinsulinemia and insulin resistance [24]; however, considering the link between other composition parameters and anthropometric measurements, we would expect that hyperinsulinemia would also be associated with other body composition markers due to insulin’s anabolic function [25], which was not the case.

Out of the thyroid hormones, only TSH showed a connection to BCM, FFM, ECW and TBW. It should be emphasized that obese children can have mildly elevated but still in normal range TSH levels, which can be considered a potential marker of metabolic risk factors in obese children [26]. However, the association with FM in our research group was not confirmed.

In biochemical laboratory results, associations were found between all body composition parameters and urate. The latter serves as a biomarker for the diagnosis of essential hypertension [27]. Hyperuricemia has also been associated with renal dysfunction, albuminuria and obesity [28]; the latter association has also been confirmed by our study. Creatinine levels correlated with all body composition parameters. The ratio between creatinine and albumin in the urine was associated only with FM. On the contrary, the proteins in the 24 h urine collection and all body composition parameters correlated, except FM and PA. Our associations are due to the known associations between obesity and kidney function, where obesity is associated with glomerular hyperperfusion and hyperfiltration [29], which can be apparently associated with body composition.

The majority of the liver enzymes correlated with body composition parameters, except aspartate aminotransferase (AST). Additionally, bilirubin was associated with almost all body composition parameters, but not PA and FM. Non-alcoholic fatty liver disease has now been recognized as the most common cause of abnormal liver function tests [30], and in our cohort, the association between FM and hepatic steatosis has also been confirmed, as indicated below. Both AST and ALT suggest liver cell injury; however, AST is produced by other organs and tissues. Therefore, ALT is considered to be more liver-specific. It has been suggested that ALT correlates with the degree of abdominal adiposity [31]. In recent studies, adiposity in children has more association with increased ALT and to a lesser degree with GGT [32], which supports our findings. However, both ALT and GGT correlated significantly with FM even after Bonferroni correction.

Homocysteine correlated with body composition parameters, except with PA and FM, which is unexpected since homocysteine concentrations were significantly elevated among obese patients [33]. This indicates that obesity might be associated with homocysteine by another mechanism and not mainly by fat mass.

Next, there were significant correlations of BIA with triglycerides, HDL cholesterol (except with PA) and apolipoprotein A1 (except with PA), as well as of FM with both LDL cholesterol and apolipoprotein B. Fats in the blood have traditionally been associated with obesity [34], which was confirmed by BIA measurement and for some new atherosclerotic risk factors [35], such as apolipoprotein A1. The correlation between FM and HDL cholesterol remained even after Bonferroni correction. Based on our study, there is no proven association between lipoprotein (a) and body composition parameters.

Vitamin D correlated negatively with FM, while B12, folates and trace elements did not show any association with either FM or FFM. Low vitamin D levels indicate fewer outdoor activities in children with obesity, where a sedentary lifestyle is a risk factor [36].

The body composition parameters were different between boys and girls except for FM. The group with hepatic steatosis had a higher mean FM measurement, which is in accordance with other studies [37]. The left ventricular hypertrophy group had significantly higher body composition parameters, indicating the importance of cardiovascular risk evaluation. [38]. Amongst the group of patients with a pathological oral glucose tolerance test, body composition parameters did not differ significantly, which might be the result of the small cohort. Higher insulin levels were much more common, showing the correlation with FM.

The main limitation of our study was the small cohort of patients. In addition, many variables were used that could be further investigated separately, and some variables were collinear. Associations found could be due to potential confounding factors, such as age, gender or other risk factors, that were not included. This has also been demonstrated after using Bonferroni correction, where correlations were less pronounced.

## 5. Conclusions

In children with hypertension, FM correlated significantly with insulin levels, urate, serum creatinine, lipid fractions, vitamin D and microalbuminuria as well with hepatic steatosis, systolic blood pressure and left ventricular hypertrophy. Our study shows that in addition to anthropometrical measurements and nutritional evaluation, BIA could be an important method for early and non-invasive cardiovascular risk determination in children. Further studies investigating different risk groups of children as well as healthy populations are needed to confirm our findings.

## Figures and Tables

**Table 1:** Descriptive statistics of the researched cohort; BMI—body mass index, WC—waist circumference, HC—hip circumference, PA—phase angle, BCM—body cell mass, FFM—fat-free mass, FM—fat mass, TBW—total body water, ECW—extracellular water, IMT—intima media thickness, TSH—thyroid stimulating hormone, T3—triiodothyronine, T4—thyroxine, AST—aspartate aminotransferase, ALT—alanine aminotransferase, GGT—gama glutamil transferase, A/C—albumin/creatinine.

Variable	Boys (N = 120)	Girls (N = 86)	Normal Weight (N = 111)	Overweight (N = 95)
**Age (years)**	15 ± 7	12 ± 8	13 ± 8	14 ± 6
**Height (cm)**	170 ± 32 *	157 ± 32 *	158 ± 40 *	164 ± 24 *
**Weight (kg)**	69 ± 39 *	49 ± 35 *	43 ± 34 *	77 ± 33 *
**BMI (kg/m^2^)**	24 ± 8 *	20.4 ± 8.8 *	18.2 ± 5.2 *	27 ± 7.1 *
**WC (cm)**	85 ± 23 *	73 ± 21 *	73 ± 17 *	93 ± 22 *
**HC (cm)**	98 ± 21 *	86 ± 28 *	86 ± 23.5 *	104 ± 24 *
**PA (°)**	6.5 ± 1.4 *	6 ± 1.2 *	6 ± 1.4 *	6.5 ± 1.5 *
**BCM (kg/m)**	28.7 ± 20 *	20 ± 15.3 *	20 ± 16.5 *	28.2 ± 17.6 *
**FFM (kg/m)**	51 ± 30.5 *	40 ± 22.3 *	38 ± 26.6 *	51.4 ± 25 *
**FM (kg/m)**	13.8 ± 15.4	12.8 ± 14.6	9.2 ± 7.6 *	23.5 ± 16.7 *
**TBW (L/m)**	38.3 ± 18.9 *	30 ± 16.7 *	30 ± 19 *	38.3 ± 14.9 *
**ECW (L/m)**	15.7 ± 8.6 *	12.7 ± 5.9 *	12.7 ± 7.4 *	15.6 ± 7.4 *
**Systolic pressure (mmHg)**	123 ± 17 *	115 ± 15 *	115 ± 17 *	123 ± 16 *
**Diastolic pressure (mmHg)**	68 ± 11	68.5 ± 11	68 ± 9	68 ± 10
**Pulse wave velocity (m/s)**	6.6 ± 1.9	6.4 ± 2.2	6.6 ± 1.9	6.6 ± 2.2
**Right IMT (mm)**	0.46 ± 0.12	0.46 ± 0.11	0.46 ± 0.11	0.46 ± 0.11
**Left IMT (mm)**	0.46 ± 0.1	0.45 ± 0.1	0.47 ± 0.08	0.45 ± 0.1
**Insulin at 0 h (mU/L)**	18.7 ± 16.7	20.8 ± 12.1	10.9 ± 7.13 *	19.9 ± 15.6 *
**Insulin at 2 h (mU/L)**	91 ± 97 *	149 ± 173 *	25.66 ± 38.3 *	121.5 ± 126 *
**TSH (mU/L)**	2.3 ± 1.4	2.5 ± 1.6	2.5 ± 1.4	2.18 ± 1.5
**T3 (pmol/L)**	6.1 ± 1.2 *	5.5 ± 1.19 *	5.7 ± 1	6.1 ± 1.35
**T4 (pmol/L)**	15.4 ± 2.7 *	14.7 ± 2.8 *	15.3 ± 3.2	15.2 ± 2.8
**Leukocytes (×10^9^)**	6.3 ± 2.1 *	7.1 ± 2.2 *	6.25 ± 1.8 *	7 ± 2.3 *
**Erythrocytes (×10^9^)**	5.2 ± 0.6 *	4.8 ± 0.5 *	4.9 ± 0.6 *	5.1 ± 0.5 *
**Hemoglobin (g/L)**	148 ± 22 *	133 ± 12 *	135 ± 22 *	141 ± 19 *
**Mean corpuscular volume (fl)**	82 ± 6.7	82.5 ± 6	82.9 ± 6.6	82 ± 6.2
**Thrombocytes (×10^9^)**	280 ± 92	298 ± 86	277 ± 96 *	300 ± 77 *
**Glucose (mmol/L)**	4.7 ± 0.6	4.7 ± 0.6	4.7 ± 0.5	4.7 ± 0.6
**Urea (mmol/L)**	4.7 ± 1.4 *	3.9 ± 1.1 *	4.3 ± 1.4	4.4 ± 1.4
**Creatinine (mmol/L)**	65 ± 30 *	54 ± 20 *	58 ± 24	59 ± 31
**Urate (µmol/L)**	323 ± 113 *	274 ± 110 *	250 ± 113 *	326 ± 101 *
**C reactive protein (mg/L)**	3 ± 0	3 ± 1	3 ± 0	3 ± 1
**Total bilirubin (µmol/L)**	10 ± 7 *	8 ± 4 *	10.6 ± 7	9 ± 6
**Direct bilirubin (µmol/L)**	3 ± 1 *	2 ± 1 *	2.5 ± 1	2 ± 1
**AST (µkat/L)**	0.38 ± 0.2 *	0.3 ± 0.15 *	0.32 ± 0.14	0.39 ± 0.22
**ALT (µkat/L)**	0.45 ± 0.3 *	0.35 ± 0.15 *	0.35 ± 0.13 *	0.49 ± 0.34 *
**GGT (µkat/L)**	0.45 ± 0.17 *	0.37 ± 0.14 *	0.37 ± 0.09 *	0.48 ± 0.17 *
**Alkaline phosphatase (µkat/L)**	2.5 ± 2.6	2.5 ± 3	2.6 ± 3	2.5 ± 2.7
**Total cholesterol (mmol/L)**	4.5 ± 1.1	4.7 ± 1.2	4.7 ± 1.3	4.55 ± 1
**Triglycerides (mmol/L)**	1 ± 0.6	1.1 ± 0.6	0.8 ± 0.5	1.15 ± 0.6
**HDL cholesterol (mmol/L)**	1.4 ± 0.4	1.4 ± 0.4	1.6 ± 0.5 *	1.3 ± 0.33 *
**LDL cholesterol (mmol/L)**	2.7 ± 1.2	3 ± 1.1	2.6 ± 1.4	2.8 ± 1
**Cystatin C (mg/L)**	0.87 ± 0.14	0.89 ± 0.17	0.88 ± 0.17	0.87 ± 0.13
**Lipoprotein (a) (mg/dL)**	53 ± 135	83 ± 344	73 ± 221	58 ± 133
**Apolipoprotein A1 (g/L)**	1.4 ± 0.2	1.48 ± 0.23	1.46 ± 0.26	1.4 ± 0.23
**Apolipoprotein B (g/L)**	0.72 ± 0.27	0.8 ± 0.32	0.69 ± 0.3	0.79 ± 0.25
**Urine A/C ratio**	8 ± 5 *	16 ± 46 *	10.5 ± 32 *	8 ± 11 *
**Proteins in 24 h urine (g/day)**	0.18 ± 0.1 *	0.14 ± 0.08 *	0.18 ± 0.1	0.16 ± 0.11
**Homocysteine (µmol/L)**	8.9 ± 3.2 *	7.9 ± 2.5 *	8.1 ± 2.4 *	8.9 ± 3.1 *
**Vitamin D (nmol/L)**	66.5 ± 41.5	61.2 ± 27.2	69.2 ± 47.1	61.6 ± 33.7
**Vitamin B12 (pmol/L)**	342 ± 139	350 ± 217	407 ± 154 *	329 ± 126 *
**Folates (nmol/L)**	14.5 ± 7.1	18.5 ± 16.8	15.7 ± 7.9	15.6 ± 10.8
**Zinc (µmol/L)**	17.9 ± 4.1	16.9 ± 4.7	16.3 ± 6.2	18.7 ± 3.6
**Selenium (µmol/L)**	75.7 ± 24.6	66.8 ± 4.9	71.5 ± 21.2	74.5 ± 24.6

*—statistically significant difference.

**Table 2:** Correlations described with Pearson correlation factor (r) and *p*-value between clinical and body composition parameters; PA—phase angle, BCM—body cell mass, FFM—fat-free mass, FM—fat mass, TBW—total body water, ECW—extracellular water, BMI—body mass index, WC—waist circumference, HC—hip circumference, IMT—intima media thickness, TSH—thyroid stimulating hormone, T3—triiodothyronine, T4—thyroxine, AST—aspartate aminotransferase, ALT—alanine aminotransferase, GGT—gama glutamil transferase, A/C—albumin/creatinine.

	PA	BCM	FFM	FM	TBW	ECW
Height	r = 0.296 *p* < 0.001 *	r = 0.855 *p* < 0.001	r = 0.888 *p* < 0.001 *	r = 0.464 *p* < 0.001	r = 0.895 *p* < 0.001 *	r = 0.899 *p* < 0.001
Weight	r = 0.318 *p* < 0.001 *	r = 0.874 *p* < 0.001 *	r = 0.890 *p* < 0.001 *	r = 0.819 *p* < 0.001	r = 0.878 *p* < 0.001	r = 0.841 *p* < 0.001 *
BMI	r = 0.308 *p* < 0.001 *	r = 0.686 *p* < 0.001	r = 0.689 *p* < 0.001	r = 0.903 *p* < 0.001 *	r = 0.675 *p* < 0.001	r = 0.616 *p* < 0.001 *
WC	r = 0.210 *p* = 0.015	r = 0.679 *p* < 0.001 *	r = 0.694 *p* < 0.001	r = 0.821 *p* < 0.001	r = 0.693 *p* < 0.001 *	r = 0.646 *p* < 0.001
HC	r = 0.181 *p* = 0.051	r = 0.729 *p* < 0.001 *	r = 0.738 *p* < 0.001	r = 0.783 *p* < 0.001	r = 0.732 *p* < 0.001	r = 0.703 *p* < 0.001
Systolic pressure	r = 0.311 *p* < 0.001 *	r = 0.594 *p* < 0.001	r = 0.590 *p* < 0.001	r = 0.340 *p* < 0.001	r = 0.582 *p* < 0.001 *	r = 0.534 *p* < 0.001
Diastolic pressure	r = 0.035 *p* = 0.631	r = 0.050 *p* = 0.495	r = 0.053 *p* = 0.472	r = 0.014 *p* = 0.845	r = 0.048 *p* = 0.517	r = 0.013 *p* = 0.859
Pulse wave velocity	r = 0.017 *p* = 0.865	r = 0.266 *p* = 0.008	r = 0.275 *p* = 0.005	r = 0.095 *p* = 0.344	r = 0.269 *p* = 0.007	r = 0.239 *p* = 0.016
Right IMT	r = 0.112 *p* = 0.228	r = 0.245 *p* = 0.007	r = 0.226 *p* = 0.013	r = 0.050 *p* = 0.592	r = 0.216 *p* = 0.018	r = 0.204 *p* = 0.026
Left IMT	r = 0.076 *p* = 0.413	r = 0.215 *p* = 0.019	r = 0.195 *p* = 0.034	r = 0.119 *p* = 0.196	r = 0.185 *p* = 0.044	r = 0.190 *p* = 0.038
Insulin at 0 h	r = 0.027 *p* = 0.795	r = 0.206 *p* = 0.048	r = 0.238 *p* = 0.021	r = 0.422 *p* < 0.001	r = 0.252 *p* = 0.014	r = 0.177 *p* = 0.088
Insulin at 2 h	r = −0.127 *p* = 0.276	r = −0.050 *p* = 0.666	r = 0.008 *p* = 0.948	r = 0.344 *p* = 0.002	r = 0.019 *p* = 0.872	r = −0.041 *p* = 0.720
TSH	r = 0.024 *p* = 0.798	r = −0.208 *p* = 0.028	r = −0.209 *p* = 0.026	r = −0.002 *p* = 0.980	r = −0.214 *p* = 0.023	r = −0.219 *p* = 0.020
T3	r = −0.058 *p* = 0.595	r = 0.000 *p* = 0.998	r = 0.029 *p* = 0.726	r = −0.051 *p* = 0.595	r = 0.026 *p* = 0.791	r = 0.071 *p* = 0.461
T4	r = 0.081 *p* = 0.403	r = −0.060 *p* = 0.540	r = −0.107 *p* = 0.267	r = −0.127 *p* = 0.189	r = −0.123 *p* = 0.203	r = −0.112 *p* = 0.246
Glucose	r = 0.011 *p* = 0.897	r = −0.035 *p* = 0.677	r = −0.044 *p* = 0.599	r = 0.009 *p* = 0.914	r = −0.029 *p* = 0.734	r = −0.051 *p* = 0.541
Urea	r = 0.037 *p* = 0.640	r = 0.038 *p* = 0.638	r = 0.015 *p* = 0.853	r = −0.086 *p* = 0.282	r = 0.006 *p* = 0.939	r = 0.000 *p* = 0.999
Creatinine	r = 0.214 *p* = 0.006	r = 0.735 *p* < 0.001 *	r = 0.729 *p* < 0.001	r = 0.223 *p* = 0.004	r = 0.705 *p* < 0.001 *	r = 0.720 *p* < 0.001
Urate	r = 0.169 *p* = 0.058	r = 0.607 *p* < 0.001	r = 0.611 *p* < 0.001	r = 0.355 *p* < 0.001	r = 0.594 *p* < 0.001 *	r = 0.548 *p* < 0.001
Creactive protein	r = −0.040 *p* = 0.626	r = −0.116 *p* = 0.154	r = −0.113 *p* = 0.163	r = −0.058 *p* = 0.476	r = −0.098 *p* = 0.227	r = −0.110 *p* = 0.174
Total bilirubin	r = 0.078 *p* = 0.287	r = 0.300 *p* < 0.001 *	r = 0.258 *p* = 0.001	r = 0.018 *p* = 0.822	r = 0.230 *p* = 0.004	r = 0.341 *p* < 0.001 *
Direct bilirubin	r = 0.116 *p* = 0.156	r = 0.299 *p* < 0.001 *	r = 0.260 *p* = 0.001	r = −0.064 *p* = 0.436	r = 0.231 *p* = 0.004	r = 0.316 *p* < 0.001 *
AST	r = 0.017 *p* = 0.838	r = 0.033 *p* = 0.688	r = 0.030 *p* = 0.710	r = −0.067 *p* = 0.407	r = 0.023 *p* = 0.777	r = 0.022 *p* = 0.782
ALT	r = 0.141 *p* = 0.080	r = 0.334 *p* < 0.001 *	r = 0.315 *p* < 0.001 *	r = 0.328 *p* < 0.001 *	r = 0.305 *p* < 0.001 *	r = 0.258 *p* = 0.001
GGT	r = 0.189 *p* = 0.019	r = 0.441 *p* < 0.001	r = 0.421 *p* < 0.001	r = 0.406 *p* < 0.001 *	r = 0.413 *p* < 0.001	r = 0.351 *p* < 0.001 *
Alkaline phosphatase	r = −0.228 *p* = 0.020	r = −0.539 *p* < 0.001	r = −0.526 *p* < 0.001	r = −0.282 *p* = 0.004	r = −0.484 *p* < 0.001 *	r = −0.505 *p* < 0.001
Total cholesterol	r = −0.102 *p* = 0.236	r = −0.155 *p* = 0.070	r = −0.178 *p* = 0.084	r = 0.098 *p* = 0.253	r = −0.134 *p* = 0.118	r = −0.134 *p* = 0.117
Triglycerides	r = 0.016 *p* = 0.848	r = 0.234 *p* = 0.006	r = 0.247 *p* = 0.004	r = 0.465 *p* < 0.001	r = 0.248 *p* = 0.003	r = 0.311 *p* < 0.001 *
HDL cholesterol	r = −0.085 *p* = 0.329	r = −0.322 *p* < 0.001 *	r = −0.330 *p* < 0.001 *	r = −0.392 *p* < 0.001 *	r = −0.337 *p* < 0.001 *	r = −0.335 *p* < 0.001 *
LDL cholesterol	r = −0.061 *p* = 0.481	r = −0.076 *p* = 0.383	r = −0.072 *p* = 0.402	r = 0.181 *p* = 0.035	r = −0.057 *p* = 0.513	r = −0.066 *p* = 0.44
Cystatin C	r = −0.010 *p* = 0.920	r = 0.093 *p* = 0.340	r = 0.113 *p* = 0.241	r = −0.019 *p* = 0.846	r = 0.115 *p* = 0.234	r = 0.135 *p* = 0.162
Lipoprotein (a)	r = 0.140 *p* = 0.122	r = −0.072 *p* = 0.430	r = −0.113 *p* = 0.209	r = −0.069 *p* = 0.447	r = −0.097 *p* = 0.283	r = −0.141 *p* = 0.118
Apolipoprotein A1	r = −0.084 *p* = 0.373	r = −0.335 *p* < 0.001 *	r = −0.340 *p* < 0.001 *	r = −0.222 *p* = 0.016	r = −0.351 *p* < 0.001 *	r = −0.347 *p* < 0.001 *
Apolipoprotein B	r = −0.026 *p* = 0.786	r = 0.041 *p* = 0.662	r = 0.049 *p* = 0.605	r = 0.278 *p* = 0.003	r = 0.063 *p* = 0.503	r = 0.044 *p* = 0.640
Urine A/C ratio	r = −0.130 *p* = 0.175	r = −0.151 *p* = 0.114	r = −0.126 *p* = 0.186	r = −0.204 *p* = 0.031	r = −0.112 *p* = 0.241 *	r = −0.085 *p* = 0.374 *
Proteins in 24 h urine	r = 0.121 *p* = 0.166	r = 0.404 *p* < 0.001 *	r = 0.414 *p* < 0.001	r = −0.020 *p* = 0.823	r = 0.408 *p* < 0.001	r = 0.402 *p* < 0.001
Homocysteine	r = 0.065 *p* = 0.486	r = 0.307 *p* = 0.001	r = 0.294 *p* = 0.001	r = 0.088 *p* = 0.642	r = 0.276 *p* = 0.002	r = 0.328 *p* < 0.001
Vitamin D	r = −0.157 *p* = 0.104	r = −0.095 *p* = 0.324	r = −0.071 *p* = 0.459	r = −0.282 *p* = 0.003	r = −0.061 *p* = 0.524	r = −0.028 *p* = 0.768
Vitamin B12	r = 0.192 *p* = 0.328	r = 0.169 *p* = 0.390	r = 0.150 *p* = 0.438	r = −0.307 *p* = 0.105	r = 0.148 *p* = 0.444	r = 0.032 *p* = 0.868
Folates	r = −0.291 *p* = 0.150	r = −0.354 *p* = 0.076	r = −0.299 *p* = 0.130	r = −0.153 *p* = 0.447	r = −0248 *p* = 0.213	r = −0.464 *p* = 0.015
Zinc	r = 0.322 *p* = 0.134	r = 0.214 *p* = 0.327	r = 0.142 *p* = 0.508	r = 0.414 *p* = 0.044	r = 0.129 *p* = 0.549	r = 0.074 *p* = 0.730
Selenium	r = 0.130 *p* = 0.555	r = 0.335 *p* = 0.118	r = 0.428 *p* = 0.037	r = −0.125 *p* = 0.561	r = 0.473 *p* = 0.020	r = 0.187 *p* = 0.382

*—statistically significant difference after Bonferroni correction.

**Table 3:** The independent samples t-test results presented as mean ± standard deviation and *p*-value; PA—phase angle, BCM—body cell mass, FFM—fat-free mass, FM—fat mass, TBW—total body water, ECW—extracellular water.

		PA	BCM	FFM	FM	TBW	ECW
Gender	Boys	6.7 ± 1.6	28 ± 11.3	48 ± 17	17.4 ± 12	36 ± 12	15 ± 5
	Girls	6.1 ± 1.8	20 ± 8.9	36.7 ± 13	16 ± 12	28 ± 9.9	12 ± 4
	*p*-value	0.025	<0.001	<0.001	0.415	<0.001	<0.001
Hepatic steatosis	Yes	7 ± 2.6	30 ± 9	51 ± 15	31 ± 11	39 ± 10	15.6 ± 4.5
	No	6.5 ± 1.3	25 ± 11	44 ± 17	17 ± 12	34 ± 12	14 ± 4.9
	*p*-value	0.128	0.073	0.041	<0.001	0.043	0.099
Left ventricular hypertrophy	Yes	7.3 ± 1	36 ± 7.9	60 ± 12	27 ± 13	44 ± 8.8	18 ± 3.6
	No	6.5 ± 1.9	23 ± 8.8	43 ± 14	18 ± 11	32.5 ± 10	13 ± 4.4
	*p*-value	0.016	<0.001	<0.001	0.001	<0.001	<0.001
Pathological oral glucose tolerance test	Yes	6.2 ± 0.8	26.2 ± 8.6	47.2 ± 13	27 ± 10.7	35.6 ± 9.5	14.7 ± 3.4
	No	6.9 ± 1.9	30 ± 10.5	51.5 ± 16	27 ± 12.3	38.4 ± 11	15.6 ± 4.6
	*p*-value	0.225	0.248	0.393	0.994	0.421	0.555

**Table 4:** Multiple regression analysis for fat mass (FM) and selected parameters; BMI—body mass index, ALT—alanine aminotransferase, GGT—gama glutamil transferase.

Parameter	Standardized Beta Coefficient	Significance
BMI	0.880	<0.001
ALT	0.008	0.882
GGT	−0.018	0.740
HDL cholesterol	−0.030	0.507

## Data Availability

All the data are available from the corresponding author upon reasonable request.

## References

[1] Mialich M.S., Faccioli Sicchieri J.M., Jordao Junior A.A. (2014). Analysis of body composition: A critical review of the use of bioelectrical impedance analysis. Int. J. Clin. Nutr..

[2] Barbosa-Silva M.C.G., Barros A.J.D., Wang J., Heymsfield S.B., Pierson R.N. (2005). Bioelectrical impedance analysis: Population reference values for phase angle by age and sex. Am. J. Clin. Nutr..

[3] Francisco R., Matias C.N., Santos D.A., Campa F., Minderico C.S., Rocha P., Heymsfield S.B., Lukaski H., Sardinha L.B., Silva A.M. (2020). The predictive role of raw bioelectrical impedance parameters in water compartments and fluid distribution assessed by dilution techniques in athletes. Int. J. Environ. Res. Public Health.

[4] Bosy-Westphal A., Danieslzik S., Dörhöfer R.P., Later W., Wiese S., Müller M.J. (2006). Phase angle from bioelectrical impedance analysis: Population reference values by age, sex and body mass. J. Parenter. Enteral Nutr..

[5] Hsu C.Y., Lin R., Lin Y.C., Chen J.Y., Li W.C., Lee L.A., Liu K.H., Chuang H.H. (2020). Are body composition parameters better than conventional anthropometric measures in predicting pediatric hypertension?. Int. J. Environ. Res. Public Health.

[6] Pecoraro P., Guida B., Caroli M., Trio R., Falconi S., Principato S., Pietrobelli A. (2003). Body mass index and skinfold thickness versus bioimpedance analysis: Fat mass prediction in children. Acta Diabetol..

[7] Lazzer S., Bedgoni G., Agosti F., De Col A., Mornati D., Sartorio A. (2008). Comparison of dual-energy X-ray absorptiometry, air displacement plethysmography and bioelectrical impedance analysis for the assessment of body composition in severely obese Caucasian children and adolescents. Br. J. Nutr..

[8] Pietrobelli A., Andreoli A., Cervelli V., Carbonelli M.G., Peroni D.G., De Lorenzo A. (2003). Predicting fat-free mass in children using bioimpedance analysis. Acta Diabetol..

[9] Ellulu M., Patimah I., Khaza’ai H., Rahmat A., Abed Y. (2017). Obesity and inflammation: The linking mechanism and the complications. Arch. Med. Sci..

[10] Chiolero A., Cachat F., Burnier M., Paccaud F., Bovet P. (2007). Prevalence of hypertension in schoolchildren based on repeated measurements and association with overweight. J. Hypertens..

[11] Meaume S., Benetos A., Henry O.F., Rudnichi A., Safar M.E. (2001). Aortic pulse wave velocity predicts cardiovascular mortality in subjects >70 years of age. Arterioscler. Thromb. Vasc. Biol..

[12] Kulsum-Mecci N., Goss C., Kozel B.A., Garbutt J.M., Schechtman K.B., Dharnidharka V.R. (2017). Effects of obesity and hypertension on pulse wave velocity in children. J. Clin. Hypertens..

[13] Kushne R.F., Gudivaka R., Schoeller D.A. (1996). Clinical characteristics influencing bioelectrical impedance analysis measurements. Am. J. Clin. Nutr..

[14] Akern (2014). Bodygram Plus Software Guide.

[15] Skinner A.C., Perrin E.M., Moss L.A., Skelton J.A. (2015). Cardiometabolic risks and severity of obesity in children and young adults. N. Engl. J. Med..

[16] Lugue V., Closa-Monasterolo R., Rubio-Torrents C., Zaragoza-Jordana M., Ferré N., Gispert-Liauradó M., Escribano J. (2014). Bioimpedance in 7-year old children: Validation by dual X-ray absorptiometry—Part 1: Assessment of whole body composition. Ann. Nutr. Metab..

[17] Maffeis C., Pietrobelli A., Grezzani A., Provera S., Tato L. (2001). Waist circumference and cardiovascular risk factors in prepubertal children. Obes. Res..

[18] Sant’Anna Mde S., Tinoco A.L., Rosado L.E., Sant’Ana L.F., Mello Ade C., Brito I.S., Araújo L.F., Santos T.F. (2009). Body fat assessment by bioelectrical impedance and its correlation with different anatomical sites used in the measurement of waist circumference in children. J. Pediatr..

[19] Sinha R., Fisch G., Teague B., Tamborlane W.V., Banyas B., Allen K., Savoye M., Rieger V., Taksali S., Barbetta G. (2002). Prevalence of impaired glucose tolerance among children and adolescents with marked obesity. N. Engl. J. Med..

[20] Bedinger D.H., Adams S.H. (2015). Metabolic, anabolic, and mitogenic insulin responses: A tissue-specific perspective for insulin receptor activators. Mol. Cell. Endocrinol..

[21] Drozdz D., Kwinta P., Korohoda P., Pietrzyk J.A., Drozdz M., Sancewicz-Pach K. (2009). Correlation between fat mass and blood pressure in healthy children. Pediatr. Nephrol..

[22] Burniat W., Cole T.J., Lissau I., Poskitt E. (2002). Child and Adolescent Obesity: Causes and Consequences, Prevention and Management.

[23] Wunsch R., de Sousa G., Toschke A.M., Reinehr T. (2006). Intima-media thickness in obese children before and after weight loss. Pediatrics.

[24] Galli-Tsinopoulou A., Karamouzis M., Nousia-Arvanitakis S. (2003). Insulin resistance and hyperinsulinemia in prepubertal obese children. J. Pediatr. Endocrinol. Metab..

[25] Page M.M., Johnson J.D. (2018). Mild suppression of hyperinsulinemia to treat obesity and insulin resistance. Trends. Endocrinol. Metab..

[26] Aypak C., Türedi O., Yüce A., Görpelioğlu S. (2013). Thyroid-stimulating hormone (TSH) level in nutritionally obese children and metabolic co-morbidity. J. Pediatr. Endocrinol. Metab..

[27] Yanik M., Feig D.I. (2013). Serum urate: A biomarker or treatment target in pediatric hypertension?. Curr. Opin. Cardiol..

[28] Noone D.G., Marks S.D. (2013). Hyperuricemia is associated with hypertension, obesity, and albuminuria in children with chronic kidney disease. J. Pediatr..

[29] Srivastava T. (2006). Nondiabetic consequences of obesity on kidney. Pediatr. Nephrol..

[30] Festi D., Colecchia A., Sacco T., Bondi M., Roda E., Marchesini G. (2004). Hepatic steatosis in obese patients: Clinical aspects and prognostic significance. Obes. Rev..

[31] Wang J., You D., Wang H., Yang Y., Zhang D., Lv J., Luo S., Liao R., Ma L. (2021). Association between homocysteine and obesity: A meta-analysis. J. Evid.-Based Med..

[32] Johansen M.J., Gade J., Stender S., Frithioff-Bøjsøe C., Lund M.A.V., Chabanova E., Thomsen H.S., Pedersen O., Fonvig C.E., Hansen T. (2020). The effect of overweight and obesity on liver biochemical markers in children and adolescents. J. Clin. Endocrinol. Metab..

[33] Ruhl C.E., Everhart J.E. (2010). Trunk fat is associated with increased serum levels of alanine aminotransferase in the United States. Gastroenterology.

[34] Del-Rio-Navarro B.E., Velazquez-Monroy O., Lara-Esqueda A., Violante-Ortiz R., Fanghanel G., Perez-Sanchez L., Berber A. (2008). Obesity and metabolic risks in children. Arch. Med. Res..

[35] Glowinska B., Urban M., Koput A., Galar M. (2003). New atherosclerosis risk factors in obese, hypertensive and diabetic children and adolescents. Atherosclerosis.

[36] Tremblay M.S., Colley R.C., Saunders T.J., Healy G.N., Owen N. (2001). Physiological and health implications of a sedentary lifestyle. Appl. Physiol. Nutr. Metab..

[37] Fishbein M.H., Mogren C., Gleason T., Stevens W.R. (2006). Relationship of hepatic steatosis to adipose tissue distribution in pediatric nonalcoholic fatty liver disease. J. Pediatr. Gastroenterol. Nutr..

[38] Markus M.R., Werner N., Schipf S., Siewert-Markus U., Bahls M., Baumeister S.E., Völzke H., Felix S.B., Ittermann T., Dörr M. (2017). Changes in body weight and composition are associated with changes in left ventricular geometry and function in the general population: SHIP (Study of health in Pomerania). Circ. Cardiovasc. Imaging.

